# Impact of Stone Powder Content on Corrosion Resistance in Reinforced Concrete under Stray Current and Chloride Interactions

**DOI:** 10.3390/ma17010196

**Published:** 2023-12-29

**Authors:** Yuanzhu Zhang, Xuanming Zhang, Fan Jin, Xiuyi Zhao

**Affiliations:** 1 School of Engineering, Hangzhou City University, Hangzhou 310015, China; 2 College of Civil Engineering and Architecture, Zhejiang University, Hangzhou 310058, China

**Keywords:** manufactured sand, stone powder content, concrete, stray current, chloride

## Abstract

Manufactured sand, known for its artificial production, eco-friendliness, cost-effectiveness, and sustainability, serves as an excellent alternative to natural sand. Stone powder content plays a crucial role in determining the performance of manufactured sand, significantly enhancing concrete compaction and its ability to withstand environmental degradation. This study aims to explore the feasibility of using environmentally and economically advantageous manufactured sand in the construction of subway tunnel segments in coastal areas. It investigates the impact of stone powder content on the corrosion resistance of concrete made with manufactured sand under the combined influence of chloride salts and stray currents. The analysis covers corrosion tendencies, the post-rusting performance of reinforcement bars, the resistance of concrete to chloride transport, and microstructure properties, which are assessed through electron microscope scanning and mercury compression testing. The findings indicate that the corrosion resistance of manufactured sand concrete generally surpasses that of river sand concrete. Furthermore, stone powder content within the 3–8% range leads to a denser concrete microstructure, reduced porosity, lower free-chloride ion levels, increased polarization resistance of post-corrosion reinforcements, lower corrosion current density, and reduced mass loss of reinforcing bars. This research provides valuable theoretical support for promoting the use of environmentally friendly manufactured sand concrete in subway construction projects.

## 1. Introduction

Expanding infrastructure development has triggered a growing demand for sand, but the depletion of natural sand resources and environmental concerns related to sand extraction have escalated the associated costs. To mitigate these issues, strict regulations on natural sand mining and explored alternatives have been imposed. Mechanically processed sand, produced from tailings, rocks, and even construction waste, is increasingly used in the construction industry [1]. Nevertheless, in many Chinese subway projects, concrete tunnel segments, especially in coastal areas, are rarely made with manufactured sand. Coastal areas may present challenges owing to the potential presence of groundwater rich in chlorine salts and the combined effects of stray currents from the subway systems. Previous research has extensively examined the corrosion resistance of reinforced concrete exposed to stray currents and chloride ions, particularly using river sand concrete. Stray currents have been shown to decompose cement hydration products [2], increase concrete permeability, significantly reduce the ability of cement mortar to bind chloride ions [3,4], disrupt the passivation film on steel reinforcements, and substantially raise the risk of internal steel-reinforcement corrosion [5,6,7,8]. For example, after several years of operation, the internal steel bar of the shield tunnel structure of Shanghai Metro has obviously corroded [9]. In addition, corrosion and leakage accidents occurred many times in buried pipelines parallel to electric rail transit in Shanghai [10].

A critical differentiator between manufactured sand and natural sand is the presence of stone powder. Currently, there is no uniform definition of stone powder or a consistent permissible range for its content in national specifications. Different countries employ varying definitions and limits for stone powder content. For example, China, the United States, Japan, and others define stone powder as particles below 75 μm in size, while European countries such as Britain and France define them as particles below 63 μm. In the United States, the “Standard Specification for Concrete Aggregates” (ASTM C33/C33M-18) specifies a 5–7% maximum limit for stone powder content [11]. In Japan, “Crushed Stone and Manufactured Sand for Concrete” (JIS A5005:2020) sets the limit at 7% [12]. Australia’s “Aggregates and Rock for Engineering Purposes, Part 1: Concrete Aggregates” (AS 2758.1-2014) permits an increased stone powder content, with a maximum permissible limit of up to 25% [13]. In the United Kingdom and Europe, the general standard “Aggregates for Concrete” (BS EN 12620:2013) allows for a stone powder content of 15% [14]. China’s national standard, “Construction Sand” (GB/T 14684-2022), specifies that the stone powder content must not exceed 10% [15].

Bonavetti et al. [16] discovered that stone powder can enhance the internal structure of manufactured sand concrete, accelerate early cement hydration, and boost initial cement strength without adverse effects. Nehdi et al. [17] reached similar conclusions while examining the impact of limestone powder on cement mortar. Li et al. [18] conducted a comprehensive study on stone powder with varying levels of fineness and diverse lithologies. They observed that stone powder obtained from limestone and tuff reduced the setting time. In contrast, the compressive strength of quartzite–powder–blended mortar significantly increased with an enhancement in the specific surface area of the stone powder. Enhanced stone powder fineness reduced the proportion of detrimental pores in the cement paste. Wu et al. [19] investigated the influence of stone powder content (ranging from 3% to 15%) on C80 concrete made with manufactured sand. They developed a statistical analysis method to determine the optimal stone powder admixture considering the mechanical properties of concrete. However, Ahmed et al. [20] found that increasing stone powder content led to linear declines in the compressive strength and flexural strength of manufactured sand concrete. Experiments conducted by Zhang et al. [21] revealed that for concrete with a water–cement ratio of 0.32, the optimal stone powder content is 10%. In the case of concrete with a water–cement ratio of 0.45, the optimal stone powder content should not exceed 20%, as this level results in peak seepage resistance. Xu et al. [22] conducted an extensive investigation into the influence of different stone powder types on concrete permeability, demonstrating that stone powder inclusion enhances the permeability of manufactured sand concrete. Li et al. [23] showed that in the case of low-strength manufactured sand concrete, resistance to chloride penetration increased as the stone powder content rose from 0% to 20%, but frost resistance decreased. Xie et al. [24] explored various stone powder contents from 0% to 20% and found that when the stone powder content exceeded 10%, chloride diffusion coefficients were lower than those of natural sand, and permeability was superior to that of natural sand. Thangapandi et al. [25] demonstrated that concrete with 50% artificial sand, 10% aluminum powder, and 0.5% zinc oxide instead of river sand exhibited improved resistance to seawater, acid, and sulfate erosion. Yang et al. [26] experimentally determined that the depth of reinforcement carbonation and the probability of corrosion initially decreased, then increased with the increasing proportion of stone powder, reaching a minimum carbonation depth at 7% stone powder content. Zheng et al. [27] showed that the carbonation depth of manufactured sand concrete is the smallest when the fines content is 10%. When the fines content is less than 15%, the chloride and sulfate impermeability of concrete are improved effectively.

While prior studies have predominantly focused on the mechanical properties, impermeability, and resistance to chloride and carbon dioxide erosion of concrete, there has been a noticeable lack of research dedicated to exploring the influence of stone powder content on the susceptibility of manufactured sand to stray current corrosion. This information gap has limited the widespread utilization of manufactured sand in subway construction ventures. Considering the maturity of the manufactured sand industry and the strict quality control measures in place, it is not only viable but also essential to consider the use of manufactured sand as a fine aggregate in subway tunnel-lining projects. This approach can help mitigate the overexploitation of finite river sand resources, enhance the utilization of natural resources, address the environmental pollution stemming from waste resources, and promote an economic development model characterized by resource conservation, recycling, and environmental friendliness, ultimately yielding positive ecological and economic effects.

In this study, we performed indoor stray current simulation tests to investigate the influence of stone powder content on the time-varying corrosion characteristics of reinforced concrete under the combined influence of stray current and chloride salts. This comprehensive examination covers the electrochemical properties of reinforcing bars, the rate of mass loss in corroded reinforcing bars, mechanical properties, seepage resistance of manufactured sand concrete, and microstructural features. The findings of this study can provide a scientific basis for the broader adoption of manufactured sand concrete in subway tunnel-lining projects.

## 2. Selection of Fine Aggregate

To identify manufactured sand specimens that closely mimic the properties of natural sand for subsequent testing, three commercially available manufactured sands, hereafter referred to as MSA, MSB, and MSC (Zhejiang Building Materials Group Co., Ltd., Huzhou, China), each derived from basalt, tuff, and pyrophyllite. X-ray fluorescence spectrometry revealed that the composition of the pyrophyllite manufactured sand consisted of approximately 92.668% SO_2_. River sand, designated as RS (Zhejiang Building Materials Group Co., Ltd., Huzhou, China), was employed as a control group.

### 2.1. Particle Size Distribution

**Figure 1:**
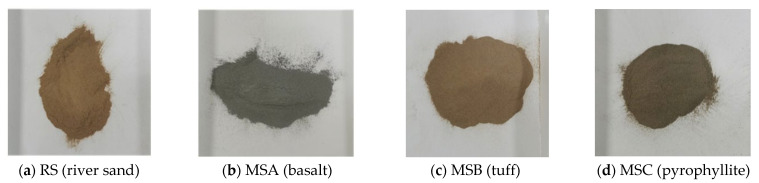
Macroscopic morphology of fine silt from fine aggregate.

**Figure 2:**
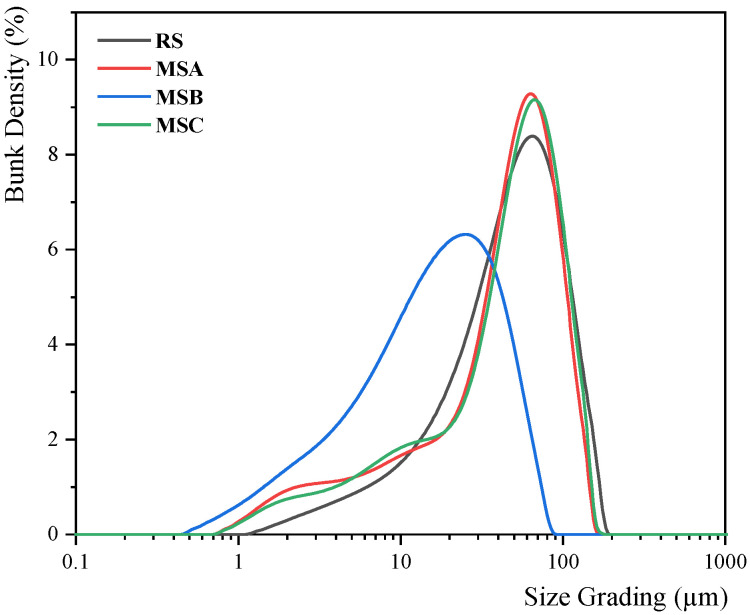
Fine aggregate particle size distribution.

Fine silt from river sand, and stone powder from each variant of manufactured sand, were sieved through a 75 μm sieve, and their morphologies are presented in Figure 1. Subsequently, a particle size distribution meter (Mastersizer 2000 model, Malvern Instruments Co., Ltd., Malvern, UK) was used to characterize the particle size distribution, as illustrated in Figure 2. Notably, the particle size distribution of the stone powder from the MSA and MSC samples closely resembles that of river sand (RS). Conversely, MSB exhibits a more uniformly distributed particle size. The median particle sizes, D_v_(50) for the various fine aggregates were 49.3, 49.7, 16.8, and 49.4 μm, respectively. The median particle sizes of MSA, MSC, and RS were nearly identical, whereas MSB exhibited a significantly different median particle size.

### 2.2. Physical Properties of Fine Aggregates

**Figure 3:**
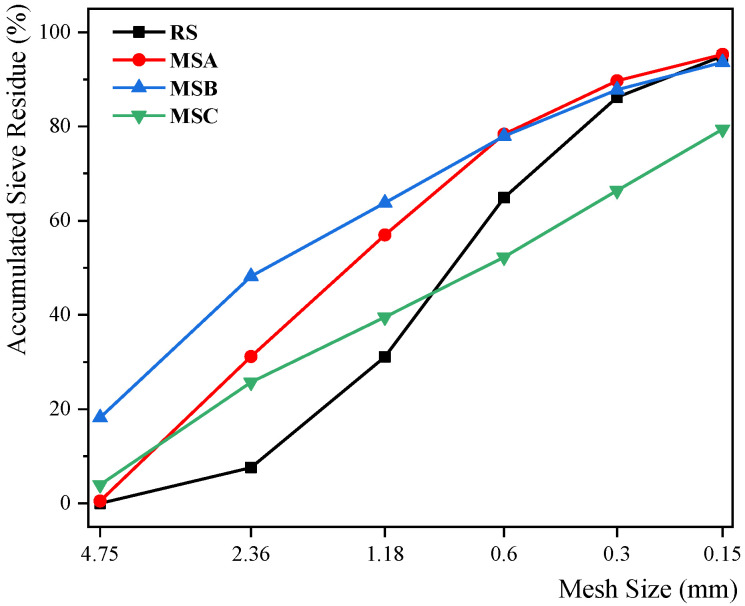
Grading curve of fine aggregate.

The assessment of the physical properties of the fine aggregates was performed in accordance with the national standard “Sand for Construction” (GB/T 14684-2022) [15]. The particle grading curves for each fine aggregate are illustrated in Figure 3. It is noteworthy that RS, MSA, and MSC all fall under Zone II gradation, whereas MSB belongs to Zone III gradation. Additionally, Table 1 provides details on other physical properties, including the fineness modulus.

Distinct differences can be observed in the physical properties of various types of manufactured sand. Notably, MSA exhibits significantly lower solidity and crushing index values compared to river sand, while MSB does not align with the grading and particle size distribution characteristics of river sand. On the other hand, MSC closely resembles river sand in terms of physical properties. Consequently, MSC was chosen as the preferred test sand for the subsequent experiments.

## 3. Indoor Testing

### 3.1. Materials

Concrete test blocks were manufactured using the mix ratio of Hangzhou Metro tunnel lining, as outlined in Table 2. The fine aggregates included manufactured sand (MSC) and river sand. Ordinary silicate cement (P.O. 42.5, Yangchun Cement Co., Ltd., Zhucheng, China) was used as the cement component, while 5–25 mm of continuous graded gravel (Zhejiang Building Materials Group Co., Huzhou, China) was employed as the coarse aggregate. The remaining materials included secondary fly ash (Platinum Refractory Co., Ltd., Gongyi, China), S95 mineral powder (Platinum Refractory Co., Ltd., Gongyi, China), tap water from the laboratory, and a highly concentrated polycarboxylic acid water-reducing agent powder (Tianjin Weihe Technology Co., Ltd., Tianjin, China) with a water-reduction rate exceeding 45%.

### 3.2. Concrete Preparation and Testing Conditions

Cubical specimens measuring 100 mm × 100 mm × 100 mm were used. In each specimen, a ϕ12 mm HRB500 rebar was embedded along the central axis of the mold to a depth of 100 mm, with 20 mm protruding above the concrete surface. The exposed portion of the rebar was securely welded to wires. After casting, the specimens were allowed to cure for 24 h. Subsequently, they were placed in a standard curing room for 28 days.

To investigate the impact of stone powder content within the manufactured sand, four specific conditions were established: 3%, 5%, 6.4% (corresponding to the initial stone powder contents of the manufactured sand samples), and 8%. These conditions were labeled SP3, SP5, SP6.4, and SP8, respectively. Additionally, a control group using river sand as the fine aggregate was designated as “RS,” as outlined in Table 3.

### 3.3. Test Methods

#### 3.3.1. Stray Current Corrosion Simulation Test

**Figure 4:**
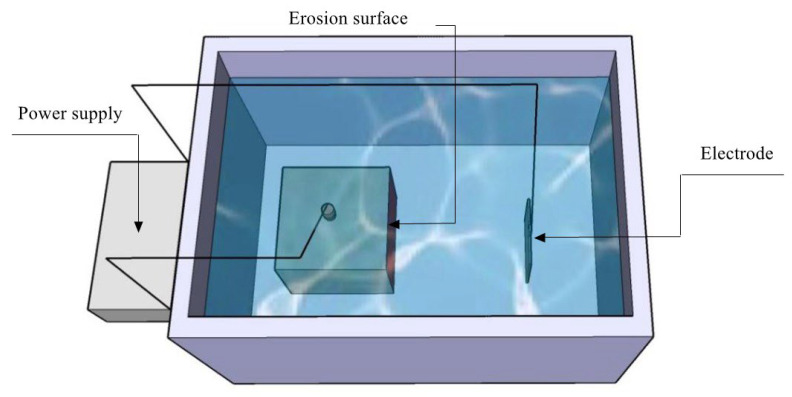
Experimental setup for simulating stray current corrosion.

This test employed a DC power supply to simulate stray current, and the test setup is illustrated in Figure 4. To conduct the test, one side of each test block was selected as the erosion surface. The remaining five surfaces were coated with epoxy resin (Ailike Co., Ltd., Dongguan, China) to seal the block. Each block representing a different condition was then immersed in a NaCl solution (Xilong Science Co., Ltd., Shantou, China) with a mass fraction of 3%, with a titanium mesh as the cathode and a steel bar in the concrete as the reactive anode, and it was energized at a constant voltage of 10 V. After energization, the test blocks were removed at 3, 7, 14, 21, and 30 days for subsequent testing. In each condition, a minimum of three blocks were employed for the tests.

#### 3.3.2. Electrochemical Testing

After the energization process, the test blocks underwent electrochemical testing using the three-electrode method [28] with an electrochemical workstation (Zennium E4 model, Zahner, Germany). In this method, the steel reinforcement within the concrete served as the working electrode. The reference electrode used was Ag–AgCl, and the auxiliary electrode was a graphite electrode. The NaCl solution was used as the corrosion solution. In the experiment, the polarization potential range applied fell within ±15 mV of the open-circuit potential of the rebar, and the scanning rate was set at 0.2 mV/s.

The corrosion rate of the metal was primarily determined using the linear polarization resistance method [29]. The linear polarization resistance (*R*_p_) for a working electrode was determined by the curve depicting the relationship between potential and electric current, then it is normalized by the surface area of the working electrode. Subsequently, the corrosion current density (*I*_corr_) of the rebar was calculated using the Stern–Geary Formula (1) [30] to assess the rate of corrosion of the rebar.

(1) $$I_{\mathrm{corr}}=\frac{B}{R_{P}}$$

In this formula, *R*_p_ represents the linear polarization resistance in Ω·cm^2^, *I*_corr_ is the corrosion current density in μA·cm^−2^, and *B* is the Stern–Geary constant. The value of B was 26 mV for reinforcement corrosion and 52 mV for reinforcement passivation. In accordance with the Broomfield criterion [31], the relationship between the corrosion current density and the degree of corrosion of the reinforcement is presented in Table 4.

#### 3.3.3. Performance Test of Corroded Rebar

Following the electrochemical test, the concrete specimen was split. Subsequently, the rebar was extracted and acid-washed to remove rust, and then it was dried and weighed. The mass loss rate (*ρ*_L_) of the rebar was calculated using Equation (2):

(2) $$\rho_{L}=\frac{m_{0}-m_{1}}{m_{0}}\times 100\%$$

In this formula, *m*_0_ represents the initial mass of the rebar in grams, and *m*_1_ is the mass of the corroded rebar after descaling and drying, also in grams. Subsequently, the tensile strength of the corroded rebar was determined using a universal testing machine in a tensile test.

#### 3.3.4. Determination of Chloride Ion Content

The Rapid Chloride Test (RCT) method is characterized by its simple operation, rapid testing, and high accuracy. A RCT–500 Rapid Chloride Test (Germann Instruments A/S Company, København, Denmark) was used to determine the free chloride ion concentration in the sample. For the concrete specimens that were damaged during splitting, powder samples were collected from depths of 1, 2, 3, and 4 cm from the invasion surface by drilling. Each time, 1.5 g of powder were weighed and added to 10 g of deionized water. The mixture was vigorously agitated, allowed to stand for 24 h to ensure complete extraction of chlorine ions, and then the concentration of free chlorine ions was determined using a rapid chloride test instrument.

#### 3.3.5. Microscopic Detection

A field-emission environmental scanning electron microscope (FEI QUANTA FEG 650 model, Thermo Fisher Scientific America, Waltham, MA, USA) was employed to examine changes in the microscopic morphology of the specimens after stray current corrosion. Samples measuring less than 15 mm × 15 mm × 15 mm after treatment were selected and subjected to pore-size distribution and porosity analysis using an AutoPore Iv 9510 high-performance fully automated mercury pressure meter (Micromeritics, Norcross, GA, USA).

## 4. Test Results and Analysis

### 4.1. Change in Polarization Resistance

The variation in the polarization resistance (*R*_p_) of the steel bars under each test condition is presented in Table 5. Initially, the *R*_p_ values for all conditions were relatively high, ranging from 1.0 × 10^6^ Ω·cm^2^ to 1.4 × 10^6^ Ω·cm^2^, indicating the excellent initial corrosion resistance of the steel bars. However, as the corrosion test progressed, the *R*_p_ values significantly decreased, signifying the corrosion effects of stray currents and chloride salts on the steel bars. After 3 days of corrosion, each group exhibited an average polarization resistance loss of approximately 97%, with *R*_p_ decreasing to the range of 2.9 × 10^4^ Ω·cm^2^ to 4.3 × 10^4^ Ω·cm^2^. This reduction indicates that the passivation film of the rebar had been compromised, leading to a substantial decline in corrosion resistance. Subsequently, the decrease in polarization resistance approaches stability around the 7-day mark. By the end of the 30-day corrosion period, the polarization resistance for each condition had decreased to approximately 2 × 10^3^ Ω·cm^2^. It indicated a shift from predominantly electrochemical polarization in the early stages to primarily concentration polarization after 7 days. Notably, the linear polarization resistance of the manufactured sand group, overall, exhibited slightly higher values compared to the river sand group. Furthermore, it was observed that as the stone powder content increased, the polarization resistance of the rebar generally increased. These observations suggest that the strategic inclusion of stone powder can enhance the corrosion resistance of the concrete structure itself.

### 4.2. Variations in Corrosion Current Density

**Figure 5:**
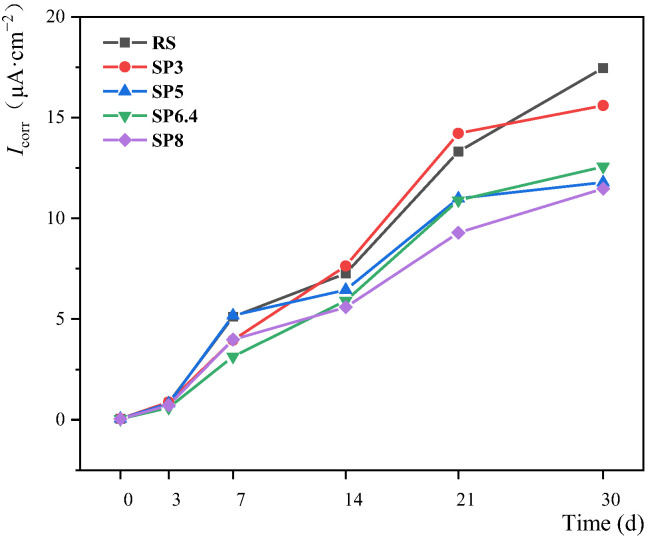
Variation of rebar *I*_corr_ with respect to stone powder content in manufactured sand.

Figure 5 depicts the time-dependent profiles of corrosion current density for each experimental condition. Prior to the corrosion test, the corrosion current density for each condition ranged from 0.04 μA·cm^−2^ to 0.05 μA·cm^−2^, indicating that the steel bars were in a passivated state. After 3 days of corrosion, the corrosion current density for each condition increased to the range of 0.6 μA·cm^−2^ to 0.9 μA·cm^−2^, signifying a moderate corrosion state. Following 7 days of corrosion, the corrosion current density for each group exceeded 1 μA·cm^−2^, indicating a heavy corrosion state. By 30 days of corrosion, the corrosion current density for each condition fell within the range of 11 μA·cm^−2^ to 16 μA·cm^−2^. Notably, the manufactured sand concrete with lower stone powder content (SP3) exhibited a corrosion current density equivalent to that of the river sand concrete. In contrast, the remaining manufactured sand concrete conditions demonstrated significantly lower corrosion current densities compared to the river sand concrete. In general, higher stone powder content was associated with lower corrosion-current density. For instance, the corrosion current density of SP8 was 11.475 μA·cm^−2^, which is 52.22%, 35.95%, 2.74%, and 9.47% less than that of RS, SP3, SP5, and SP6.4, respectively. These results indicate a clear trend of improved corrosion resistance in manufactured sand concrete as the stone powder content increases under stray current conditions.

### 4.3. Evolution of Mass Loss Rate

**Figure 6:**
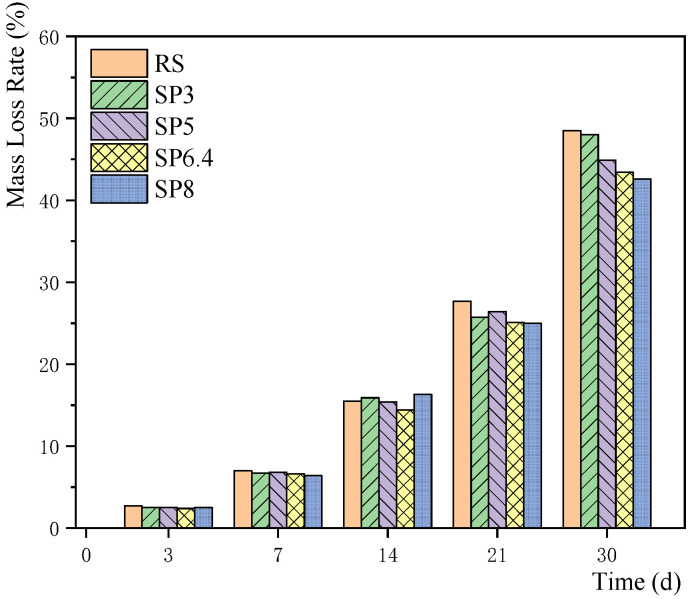
Mass loss rate of steel bars with different manufactured sand sandstone powder contents.

Figure 6 portrays the progressive changes in the mass loss rate of the rebar under different experimental conditions. Evidently, the mass loss rate of the rebar gradually increases with stray current corrosion, which highlights the adverse effects of the stray current and chloride salt environments on rebar corrosion. Combined with the corrosion current density of Section 4.2, it can be seen that the mass loss rate is about 2.5% in the medium corrosion condition on the 3rd day and about 7% in the heavy corrosion condition on the 7th day. Subsequently, the mass loss rate of reinforcement obviously increased. By Day 30, the mass loss rate surpasses 40% in all conditions. It may be mainly caused by chloride ions in solution in the later stage. In general, the mass loss rate of reinforcements in the river sand group slightly exceeds that in the manufactured sand concrete group. Furthermore, it is notable that the mass loss rate decreases significantly with higher stone powder content, emphasizing the favorable impact of stone powder content in alleviating reinforcement corrosion.

### 4.4. Tensile Strength of Corroded Steel Bars

**Figure 7:**
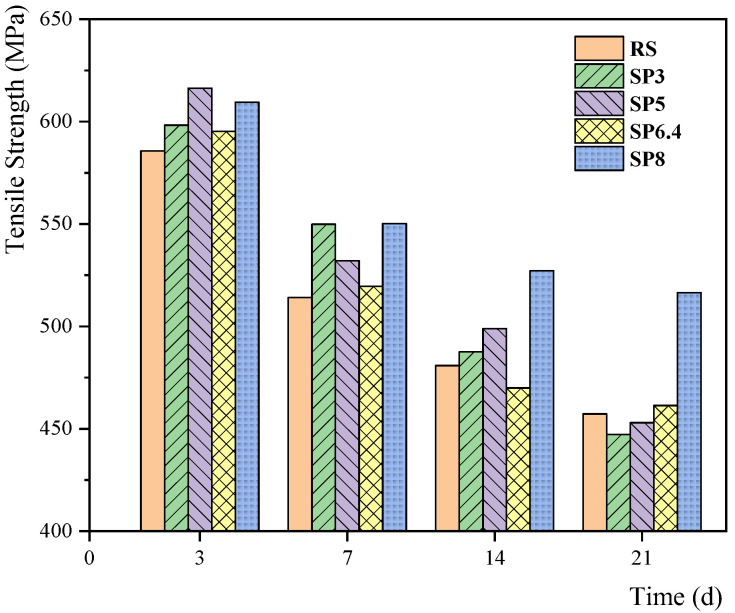
Variation in mechanical properties of steel bars corresponding to different stone powder contents.

Due to the substantial mass loss of the rebars after 30 days of stray current corrosion, we were unable to complete tensile experiments for that time point. Therefore, tensile tests were conducted for corrosion periods of 3, 7, 14, and 21 days, and the results are presented in Figure 7. It is evident that the tensile strength corresponding to each condition consistently decreases over time, with the rate of decrease gradually diminishing. The variation in tensile strength for corroded bars corresponding to different stone powder contents shows some irregularity, which is potentially associated with non-uniform corrosion patterns. Overall, the mechanical properties of rebars in the SP8 condition exhibited a significant improvement compared to other conditions during the corrosion process, highlighting the positive influence of higher stone powder content on the mechanical performance of the rebars.

### 4.5. Chloride Ion Content Variation in Concrete

**Figure 8:**
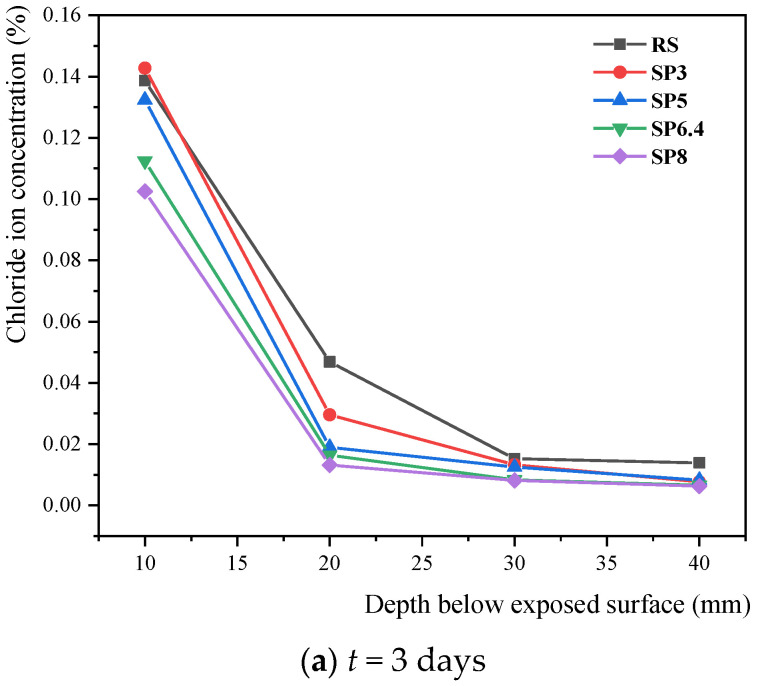

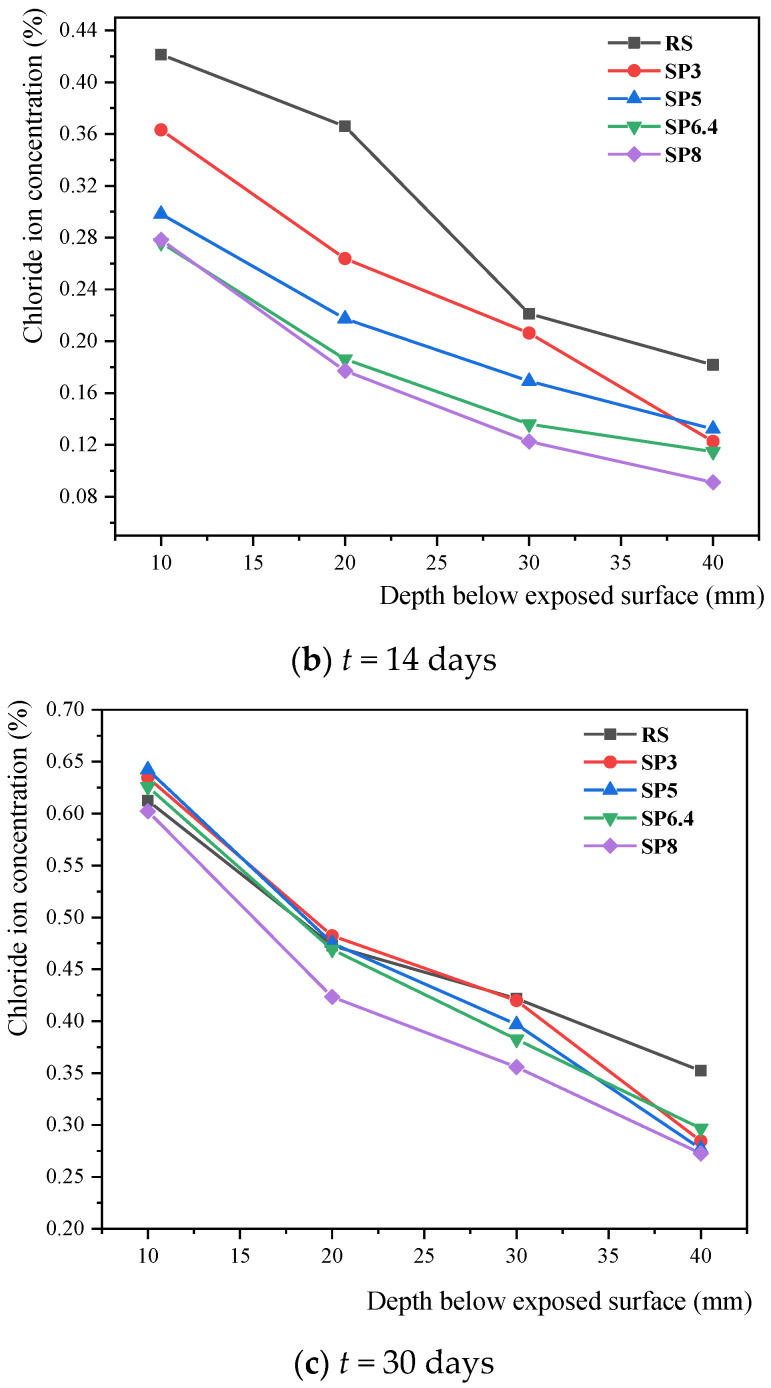
Variation of free chloride ion content in concrete with different manufactured sand powder contents.

In Figure 8, the levels of free chloride ions at various depths within the concrete specimens are presented for each condition. The figure clearly demonstrates a steady increase in free chloride ion content at each depth over time in all conditions. This signifies the continuous migration of chloride ions from the intrusion surface into the interior of the concrete. Notably, at the 3-day corrosion mark, there is a sharp decline in the concentration of free chloride ions at a depth of 20 mm from the intrusion surface, while the concentration within the range of 20 mm to 40 mm remains relatively stable. This pattern reflects the proximity of the intrusion front of external chloride ions to the intrusion surface of the specimen. Conversely, at 14 and 30 days of energization, there is a significant rise in chloride ion concentration at the considerable depth of x = 40 mm, indicating the penetration of external chloride ions deep into the concrete. Overall, the free chloride ion concentration in the river sand group tended to be higher than that in the manufactured sand group. Furthermore, it is evident that lower stone powder content is associated with higher levels of free chloride ions, indicating a reduced resistance to chloride ion transport. These findings underscore the positive influence of stone powder in enhancing resistance to chloride erosion within the studied range of stone powder content in this experiment.

### 4.6. Scanning Electron Microscopy (SEM) Analysis

**Figure 9:**
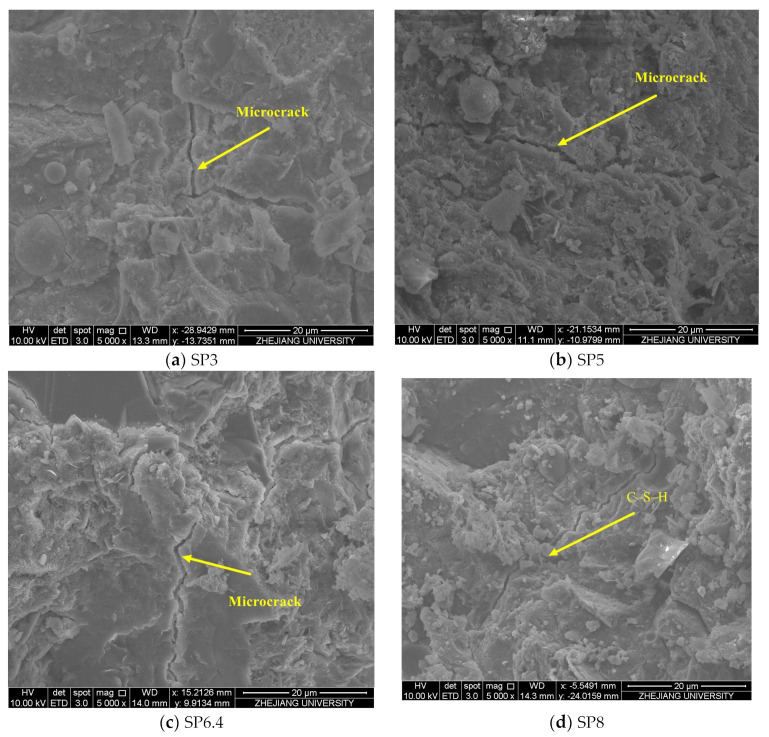
Microscopic morphology of non-corroded manufactured sand concrete under varying stone powder content conditions.

In Figure 9, scanning electron microscope images of manufactured sand concrete with varying stone powder contents are presented at 5000× magnification under conditions without stray current application. These images reveal that concrete exhibits a higher prevalence of microcracks when the stone powder content is lower. Conversely, as the stone powder content increases, both the quantity and width of these microcracks gradually decrease, resulting in an overall improved concrete structure. Notably, the microcracks in the SP8 condition are surrounded by a greater quantity of C-S-H gels, which infiltrate and fill these microcracks. This observation suggests that the optimal amount of stone powder serves a dual function, acting as both an activator and filler within the concrete, ultimately enhancing the microstructure of the concrete. These findings highlight the beneficial role played by an appropriate quantity of stone powder in improving concrete quality.

**Figure 10:**
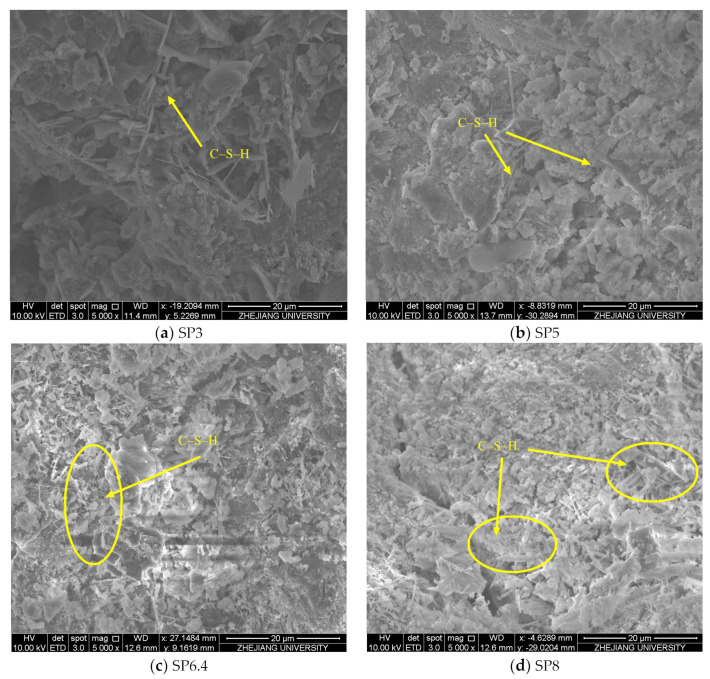
Microscopic morphology of manufactured sand concrete after corrosion for 30 days under different stone powder content conditions.

Figure 10 displays scanning electron microscope images of manufactured sand concrete with different stone powder contents, at 5000× magnification, following 30 days of stray current application. An examination of the figure reveals distinct variations in the microstructure of the concrete under different conditions. In the SP3 condition, fewer hydration products are observed, and the gaps between these products are noticeably larger, leading to a looser overall structure. Conversely, in the SP5, SP6.4, and SP8 conditions, there is a more pronounced distribution of flocculent C-S-H gel, with the quantity of C-S-H gel increasing in tandem with higher stone powder content. This suggests that conditions with greater stone powder content promote a secondary hydration reaction, generating additional C-S-H gel from products resulting from cement hydration. This process optimizes the structure of manufactured sand concrete, and its overall structural quality remains relatively superior even when exposed to stray current. However, it is worth noting that the stray current has an evident deteriorating effect on the hydration products within the concrete. This effect results in the flocculent distribution of C-S-H gel and a looser structure, ultimately leading to a decline in various properties of the concrete.

### 4.7. Mercury Intrusion Porosimetry (MIP) Analysis of Mercuric Pressure

**Figure 11:**
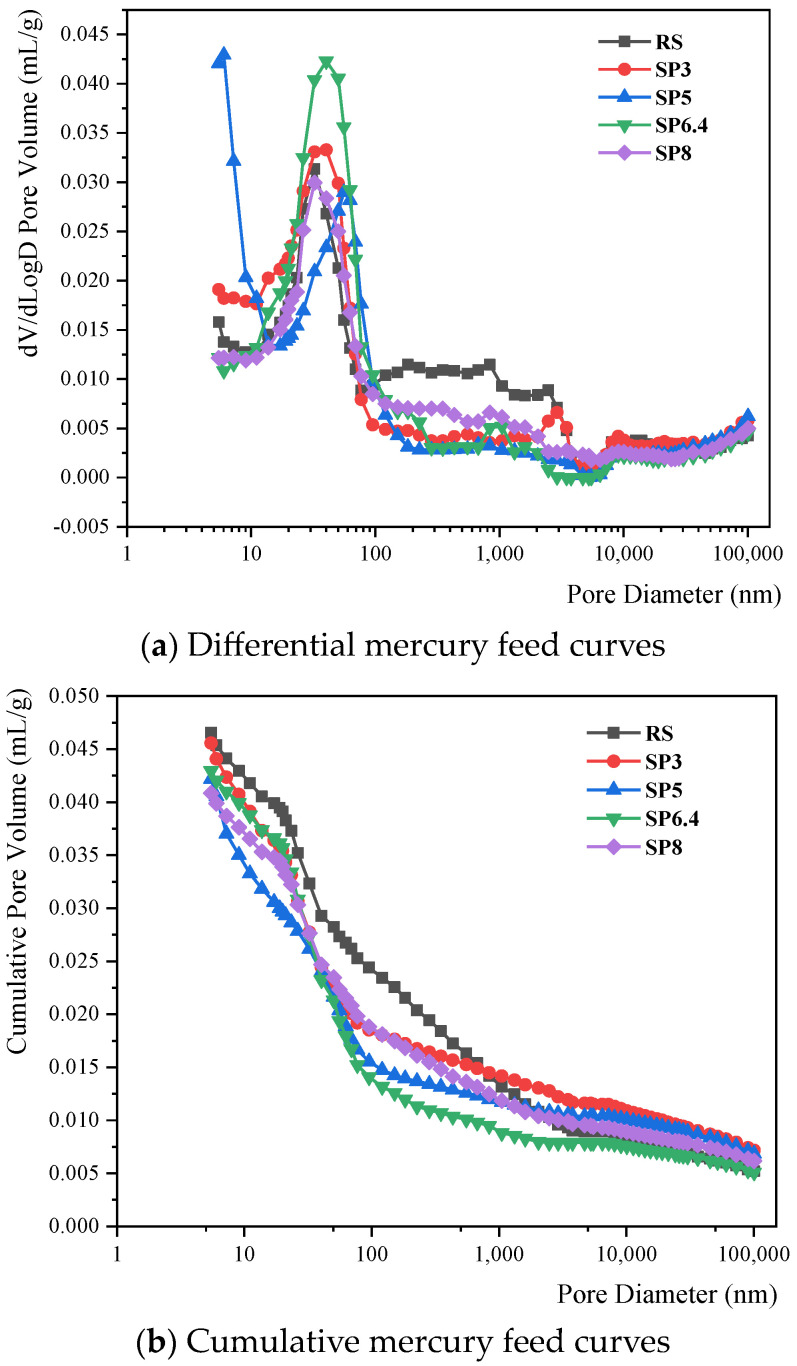
Changes in pore structure after 30 days of concrete corrosion under different manufactured sandstone powder content conditions.

Figure 11 and Table 6 provide insights into the pore structure characteristics of manufactured sand concrete subjected to 30 days of stray current corrosion, under different stone powder content conditions. Figure 11a showcases the differential mercury feed curve, with its peak denoting the most prevalent pore size, characterized by the highest probability of occurrence. In this study, the most frequently occurring pore size for each condition falls within the range of 20 nm to 50 nm. Notably, the SP5 condition displays the smallest most frequently occurring pore size. As the stone powder content increases, there is a noticeable trend towards larger overall pore sizes, particularly in the case of the smallest pore size. This observation underscores the capacity of stone powder to enhance the pore structure of the concrete microstructure. Figure 11b presents the cumulative mercury feed curve of the pore structure. Data in this figure show that the cumulative mercury feed values are relatively consistent among the various conditions, indicating comparable pore volumes within the concrete. It is noteworthy that the river sand group exhibits slightly larger pore volumes, whereas an increase in stone powder content leads to a minor reduction in mercury feed. This finding suggests that stone powder serves the role of pore filling within the concrete, ultimately strengthening its resistance to stray currents. Table 6 reveals that after 30 days of stray current corrosion, concrete porosity is highest in the river sand group, while the manufactured sand group experiences a decrease in porosity as stone powder content increases. This observation underscores the constructive influence of stone powder on the microscopic pore structure, aligning with the results of the chloride ion erosion analysis.

**Figure 12:**
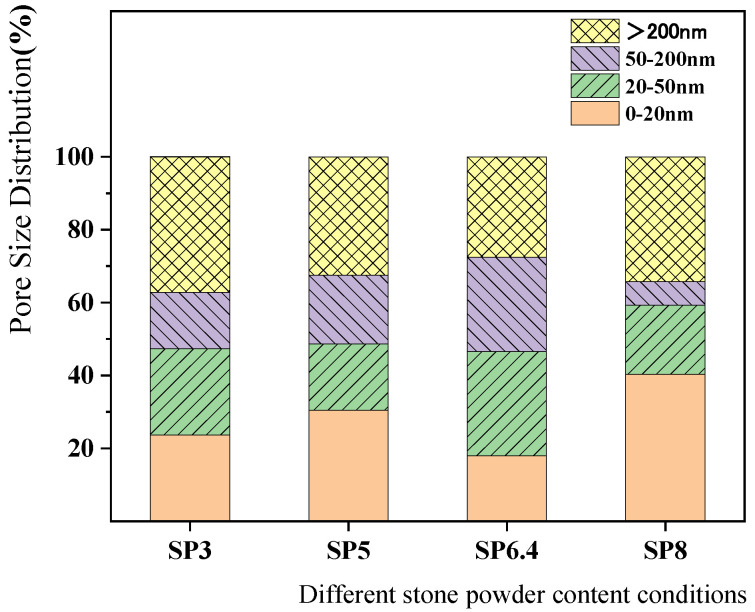
Distribution of pore sizes after 30 days of corrosion of concrete with different manufactured sand and stone powder contents.

Wu [32] introduced a pore classification system for concrete, categorizing pores based on their potential to cause harm. These categories include harmless pores (0–20 nm), less harmful pores (20–50 nm), harmful pores (50–200 nm), and more harmful pores (>200 nm). Figure 12 provides an illustration of the pore size distribution for each condition after 30 days of corrosion, using this classification system. From the figure, it is evident that the smallest harmful pore volume is observed in the SP6.4 condition. Furthermore, the SP8 condition stands out with the highest harmless pore-volume proportion. This observation highlights that when the stone powder content reaches an appropriate level, it enhances the pore structure of the concrete, resulting in improved resistance of manufactured sand concrete to stray current corrosion.

### 4.8. Discussion

The experimental results clearly indicate that the corrosion resistance of concrete in the manufactured sand group surpasses that of the river sand group. In this study, the primary component of the stone powder used in the manufactured sand concrete is SiO_2_. Notably, SiO_2_ is capable of undergoing a secondary hydration reaction with Ca(OH)_2_, a byproduct of cement hydration. This reaction results in the formation of C-S-H (hydrated calcium silicate) gel, which appears to contribute significantly to the enhanced corrosion resistance observed in manufactured sand concrete. The generation of C-S-H serves multiple purposes: It consumes the free water in the cement stone, reducing the water-permeable pores in the concrete, and, in turn, reducing the porosity and pore size of the concrete. Simultaneously, the generated C-S-H can fill the microscopic pores and cracks in the concrete, leading to improved pore structure and denser internal concrete structure. Under the combined influence of stray currents and chloride salts, chloride ions from the external environment can penetrate the concrete through diffusion and electromigration. According to Fick’s law of diffusion [33,34], the Nernst–Planck equation, and other relevant principles [35], it is clear that denser concrete results in slower chloride ion transportation, thereby enhancing resistance to rust and corrosion.

This experimental study reveals that, within the permissible range of 10% stone powder content as specified by Chinese standards, an increase in stone powder content corresponds to higher macroscopic corrosion resistance in manufactured sand concrete. The microscopic test results also support this trend, as higher stone powder content leads to the observation of more C-S-H gel, reduced porosity, higher polarization resistance of the reinforcement, lower current density, decreased mass loss of the reinforcement, increased tensile strength, and reduced concentration of free chloride ions. It is important to note that prior studies [36,37] indicate that an excessive amount of stone powder can have the opposite effect, leading to a decrease in concrete properties. This occurs when there is not enough cement paste to encapsulate coarse and fine aggregate particles. Investigating this threshold value further by expanding the range of stone powder content values is a potential area for future research.

## 5. Conclusions

This research involved the selection of manufactured sand closely resembling the properties of river sand for testing. The study focused on simulated corrosion tests of manufactured sand concrete with varying stone powder contents, which were subjected to the combined influence of stray current and chloride salts. Specifically, the investigation aimed to understand how stone powder content affects the electrochemical and mechanical properties of concrete reinforcements, as well as the chloride ion concentration and microstructure of concrete. The key findings of this study can be summarized as follows:

1. Notable variations were observed in the physical properties of different types of manufactured sand. When the physical properties of manufactured sand closely matched those of river sand, the overall corrosion resistance of the manufactured sand group outperformed that of the river sand group. This underscores the beneficial role of stone powder in enhancing the corrosion resistance of manufactured sand concrete.
2. Within the range of stone powder content values explored in this experiment, it was evident that higher stone powder content correlated with increased corrosion resistance and improved post-corrosion performance of steel reinforcements. Simultaneously, the concentration of free chloride ions within the concrete decreased as stone powder content increased. Manufactured sand containing 8% stone powder exhibited the highest polarization resistance, the lowest corrosion current, the least mass loss, and the highest post-corrosion tensile strength of the reinforcements, indicating superior resistance to chloride ion transport.
3. The SEM results indicated that prior to corrosion, both the quantity and width of microcracks in the concrete gradually decreased as the stone powder content increased. This observation suggests that an appropriate quantity of stone powder served a dual function, acting as both an activator and a filler within the concrete. This dual role contributed to the enhancement of the microstructure of concrete. However, following stray current corrosion, the structure of hydration products within the manufactured sand concrete was compromised, leading to a more porous, loose structure. This transformation underscored the erosive damage caused by stray current and chloride ion solutions to the concrete structure.
4. The results of the mercury pressure test conducted after 30 days of stray current corrosion indicated that the concrete porosity of the river sand group was the highest. In contrast, the concrete porosity of the manufactured sand group essentially decreased with the increase in stone powder content. This observation demonstrated the beneficial impact of stone powder on the microscopic pore structure of the concrete.

## Figures and Tables

**Table 1:** Fine aggregate physical properties.

Property	RS (River Sand)	MSA (Basalt)	MSB (Tuff)	MSC (Pyrophyllite)
Fineness modulus	3.40	3.51	3.33	3.43
Stone powder content (%)	-	3.65	7.31	6.42
Mud content (%)	4.44	-	-	-
MB value for methylene blue	-	0.96	1.72	0.99
Ruggedness (%)	7	4	6	6
Crushing index (%)	16	6	12	17
Apparent density (kg/m³)	2600	2730	2540	2570
Bulk density (kg/m³)	1270	1590	1450	1460
Close-packing density (kg/m³)	1400	1710	1620	1600
Void ratio	51	42	43	43

**Table 2:** Concrete mixing ratio (kg/m³).

Water	Cement	Sand	Coal Ash	Stone Powder	Coarse Aggregate	Water-Reducing Agent
158	350	730	60	30	1070	1.32

**Table 3:** Test conditions (kg/m³).

Number	Condition	River Sand	Manufactured Sand	Stone Powder	Stone Powder Content
1	SP3	365	354.05	10.95	3%
2	SP5	365	346.75	18.25	5%
3	SP6.4	365	341.64	23.36	6.4%
4	SP8	365	335.80	29.20	8%
5	RS	730	-	-	-

**Table 4:** Relationship between the degree of corrosion of steel reinforcement and corrosion current density.

Degree of Corrosion	*I*_corr_ (μA·cm^−2^)
Passivated	<0.1
Slight corrosion	0.1–0.5
Moderate corrosion	0.5–1
Severe corrosion	>1

**Table 5:** Variation in rebar *R*_p_ (Ω·cm^2^) across varying stone powder contents in manufactured sand.

Time/d	RS	SP3	SP5	SP6.4	SP8
0	1,078,397.2	1,030,480.5	1,024,407.0	1,277,245.8	1,354,802.3
3	30,849.9	29,590.7	32,097.8	43,106.2	35,950.7
7	5082.0	6567.3	5023.5	8278.9	6533.4
14	3581.5	3404.3	4030.1	4399.6	4656.0
21	1952.9	1828.5	2367.6	2386.4	2801.1
30	1489.2	1666.7	2205.5	2069.7	2265.8

**Table 6:** Pore structure characteristics after 30 days of concrete corrosion for different manufactured sand stone powder content conditions.

Working Condition	Porosity /(%)	Total Pore Capacity/(mL/g)	Total Pore Area /(m^2^/g)	Average Pore Size (nm)	Most Probable Aperture Size (nm)
SP3	9.8136	0.0464	6.093	30.44	40.71
SP5	9.5733	0.0422	7.377	22.88	29.61
SP6.4	9.3728	0.0435	5.053	34.43	40.05
SP8	8.9628	0.0414	4.434	37.30	33.02
RS	10.1359	0.0472	4.872	38.78	32.31

## Data Availability

All data, models, and code generated or used during the study appear in the submitted article.
